# Influence of Quantum-Well Width on the Electroluminescence Properties of AlGaN Deep Ultraviolet Light-Emitting Diodes at Different Temperatures

**DOI:** 10.1186/s11671-018-2756-2

**Published:** 2018-10-23

**Authors:** Shuxin Tan, Jicai Zhang, Takashi Egawa, Gang Chen, Xiangdong Luo, Ling Sun, Youhua Zhu

**Affiliations:** 1 School of Electronics and Information, Nantong University, 9 Seyuan Road, Nantong, 226019 China; 2 Department of Physics, College of Science, Beijing University of Chemical Technology, 15 East Road, Beisanhuan, Beijing, 100029 China; 3 Research Center for Nano-Device and System, Nagoya Institute of Technology, Gokiso-cho, Showa-ku, Nagoya, 466-8555 Japan; 4 Suzhou Institute of Nano-Tech and Nano-Bionics, CAS, 398 Ruoshui Road, SEID, SIP, Suzhou, 215123 China

**Keywords:** Deep ultraviolet light-emitting diodes, AlGaN, External quantum efficiency, Electroluminescence, Low temperature

## Abstract

The influence of quantum-well (QW) width on electroluminescence properties of AlGaN deep ultraviolet light-emitting diodes (DUV LEDs) was studied at different temperatures. The maximum external quantum efficiency (EQE) ratios of LED with 3.5 nm QW to that with 2 nm increased from 6.8 at room temperature (RT) to 8.2 at 5 K. However, the ratios for LED with 3.5 nm QW to that with 5 nm QW decreased from 4.8 at RT to 1.6 at 5 K. The different changes of EQE ratios were attributed to the decrease of non-radiative recombination and the increase of volume of the active region. From theoretical analysis, the LED with 2-nm wells had a shallowest barrier for electron overflow due to the quantum-confined effect, whereas the LED with 5-nm wells showed the least overlap of electron and hole due to the large internal field. Therefore, the LED with 3.5 nm QW had the highest maximum EQE at the same temperature. As temperature decreased, the current for maximum EQE decreased for all the LEDs, which was believed to be due to the increase of electron which overflowed out of QWs and the decrease of hole concentration. The results were helpful for understanding the combination of polarization effect and electron overflow in DUV LEDs.

## Background

AlGaN-based deep ultraviolet light-emitting diodes (DUV LEDs) can be widely used in the fields of solid-state lighting, medicine, biochemistry, and so on. Therefore, more and more efforts have been devoted to improve the crystal quality of the materials [1–4], the p-type doping techniques, and the optimization of the device structures [5–9]. Miyake et al. demonstrated that the AlN crystal quality can be improved significantly by high-temperature annealing [3]. By increasing the growth temperature, Sun et al. obtained high-quality AlN thick films on sapphire [2]. Recently, Jiang et al. studied the defect evolution in AlN homoepitaxial growth [1]. Their results contributed to the understanding of the AlN homoepitaxy mechanism and provided the critical techniques for improving the crystal quality. In addition, many methods were proposed to improve the light extraction, such as photonic crystals and nanostructures and surface plasmon [10–12]. In the past decades, a great progress has been obtained for this kind of LED, which was reviewed overall by Li et al. [13]. Nevertheless, the performance of the devices is still far from the practical application due to the low external quantum efficiency. It is well known that group III nitrides have wurtzite structures, in which the large spontaneous and piezoelectric fields will result in the tilted band diagram. These tilted bands had great influence on group-III nitride-based devices, such as LED, LD [14, 15], and UV detectors [16, 17]. Hirayama et al. reported the influence of quantum-well (QW) width on the photoluminescence (PL) properties in AlGaN-based single-QW DV LEDs [18]. They found that the LEDs with QW width of 1.5–1.7 nm exhibited a higher luminescence and the PL intensity decreased when the QW width was less than 1.5 nm, which was attributed to an increase of nonradiative recombination on the heterointerfaces. In this work, we fabricated DUV LEDs with different quantum-well (QW) width and studied the influence of the QW width and temperature on the electroluminescence (EL) properties. We found the LEDs with QW width of 3.5 nm exhibited the highest maximum external quantum efficiency (EQE). As the temperature decreased, the current for the maximum EQE decreased for all the LEDs, which was believed to be due to the decrease of hole concentration and the increase of overflowed electron current.

## Methods

The LEDs were grown by metal-organic chemical vapor deposition on (0001)-sapphire substrate using a 1.0-μm AlN buffer layer followed by a 0.5-μm-thick undoped Al_0.6_Ga_0.4_N and a 1.0-μm-thick n-Al_0.6_Ga_0.4_N template. The dislocation density of the template is around 6 × 10^9^ cm^− 2^ measured by transmission electron microscopy. Then Al_0.49_Ga_0.51_N/Al_0.58_Ga_0.42_N multiple QWs (MQWs) were grown as active regions. The thickness of the barriers was 5.0 nm. p-Al_0.3_Ga_0.7_N (25 nm)/Al_0.6_Ga_0.4_N (25 nm) was used as p-type layers. Finally, a 200-nm p-GaN contact layer was deposited. Based on the above structure, three samples, named LEDs A, B, and C, were grown with QW width of 2.0, 3.5, and 5.0 nm, respectively.

500 μm × 500 μm square geometry *p*-*n* junction devices were fabricated using standard lithographic techniques to define the feature and reactive ion etching to expose the *n*-Al_0.6_Ga_0.4_N Ohmic-contact layer. n-type Ohmic contacts of Ti/Al/Ni/Au (15/80/12/60 nm) were deposited by electron-beam evaporation and annealed with a rapid thermal annealing system at 900 °C for 30 s in nitrogen ambience. For transparent *p*-contacts, Ni/Au (6/12 nm) layers were electron-beam deposited and annealed in air ambience at 600 °C for 3 min. The device was completed with the deposition of Ni/Au (5/60 nm) *p* contact. The EL spectrum was measured from 5 K to room temperature (RT) using Jonin Yvon’s Symphony UV-enhanced liquid nitrogen-cooled charge-coupled device detector. To avoid the influence of thermal heating effect [19], the pulse injection with 1 μs current pulse at 0.5% was used in the EL measurements.

## Results and Discussion

**Fig. 1:**
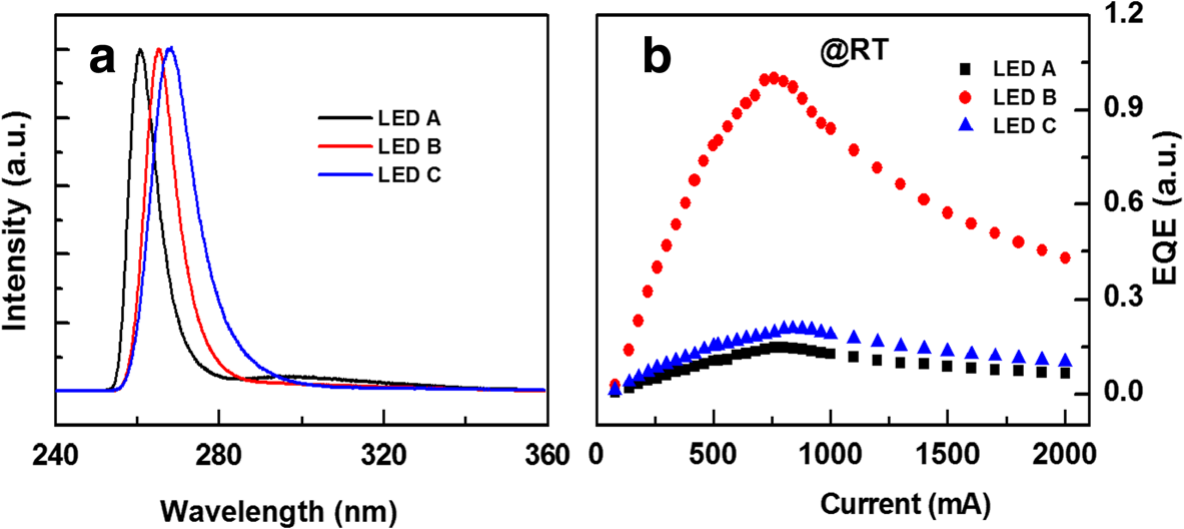
**a** The RT EL spectra for LEDs A, B, and C under direct current of 100 mA. All the spectra were normalized to the band-to-band emission. **b** The relative EQE as a function of pulse current

Figure 1a shows the EL spectra measured at room temperature (RT) for LEDs A, B, and C under direct current of 100 mA, in which all the spectra were normalized to the band-to-band emission. The EL peaks for LEDs A, B, and C were around 261, 265, and 268 nm, respectively. Obviously, the EL peak showed a redshift as the QW width increased. In addition, it should be noticed that a weak parasitic peak around 304 nm existed in the EL spectrum in LED A, which was clarified to be related with electron overflow [20]. Figure 1b shows the relative EQE as a function of pulse current for all the LEDs. All the values were normalized to the maximum EQE of LED B. The maximum EQE of LED B was about 6.8 and 4.8 times than those of LEDs A and C, respectively.

**Fig. 2:**
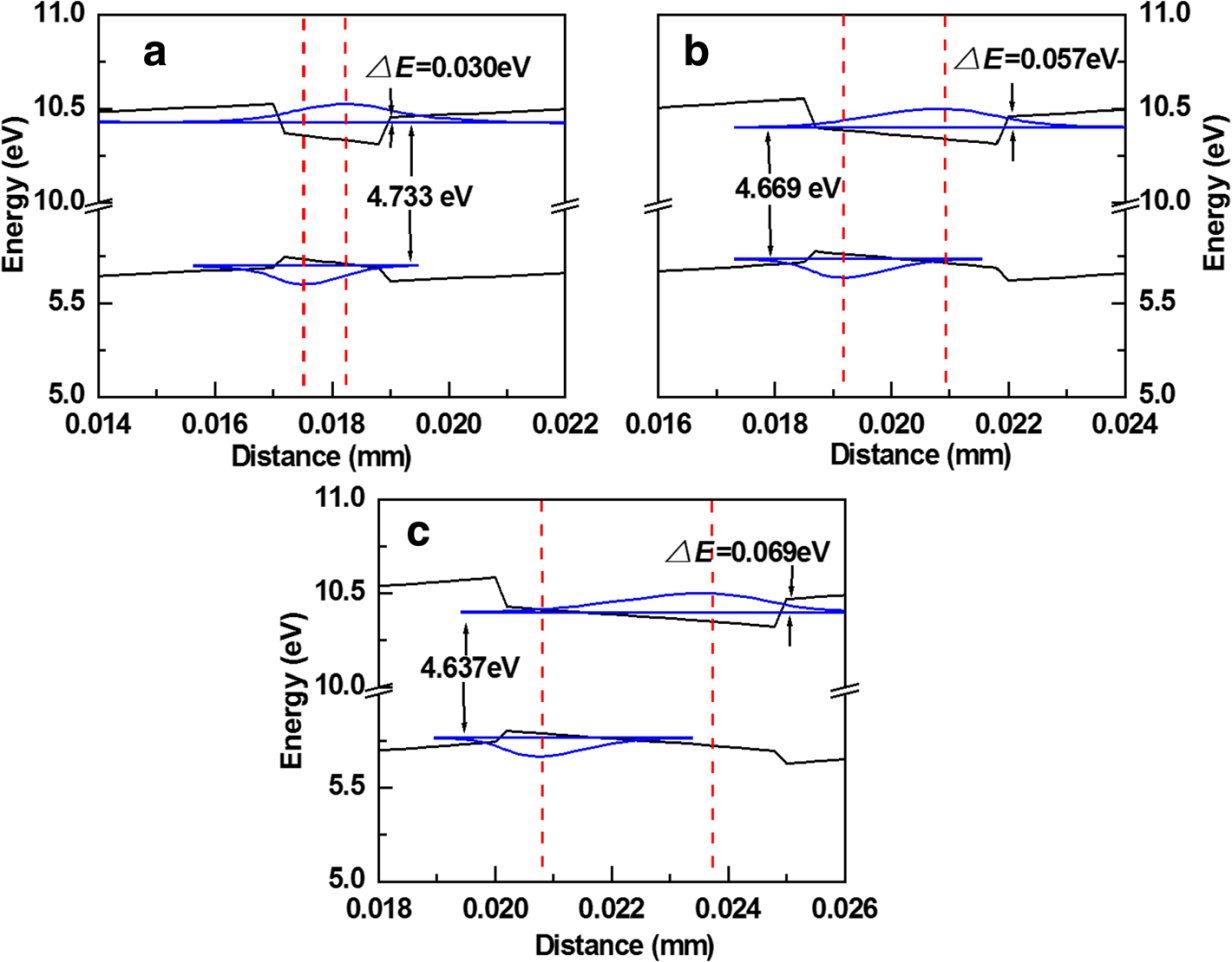
The band structure, the ground state level and carrier wave functions in one QW under current of 100 mA for (**a**) LED A, (**b**) LED B, and (**c**) LED C

In order to understand the reason, APSYS was used to simulate the energy levels and wave functions of carriers. Figure 2a–c shows the band structures, the ground state level, and carrier wave functions in one QW under current of 100 mA for LEDs A, B, and C, respectively. Due to the large internal field induced by the polarization effect and the applied forward bias, the band structure of QW showed an inclined shape and the spatial overlapping of wave functions of electrons and holes became less as the QW width increased due to the quantum-confined Stark effect (QCSE). The energy gap of the ground states for LEDs A, B, and C were 4.733, 4.669, and 4.637 eV, respectively, which coincided well with the emission wavelength as shown in Fig. 1a. In addition, it should be noticed that the confined ability of carriers by the QWs decreased as the QW width decreased. The quantum-confined effect resulted in the increase of the ground state level as the QW width decreased. The values of barrier height were 0.030, 0.057, and 0.069 eV for LEDs A, B, and C, respectively. Therefore, the EQE of LED A was less than that of LED B due to the electron current overflow, which could be confirmed by the obvious parasitic peak shown in Fig. 1a. Though LED C had the highest barrier for electron overflow in all the devices, its EQE was still less than that of LED B due to the QCSE.

**Fig. 3:**
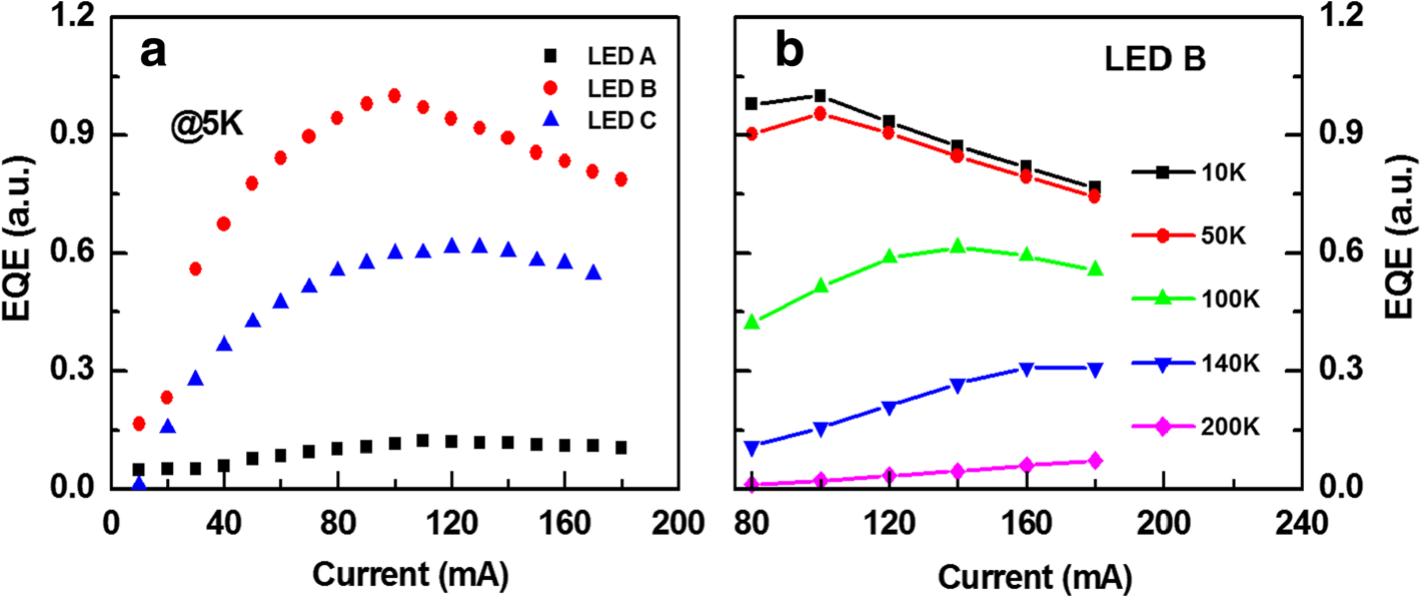
**a** The relative EQE at 5 K and (**b**) the current-dependent relative EQE at different temperature for LED B

**Fig. 4:**
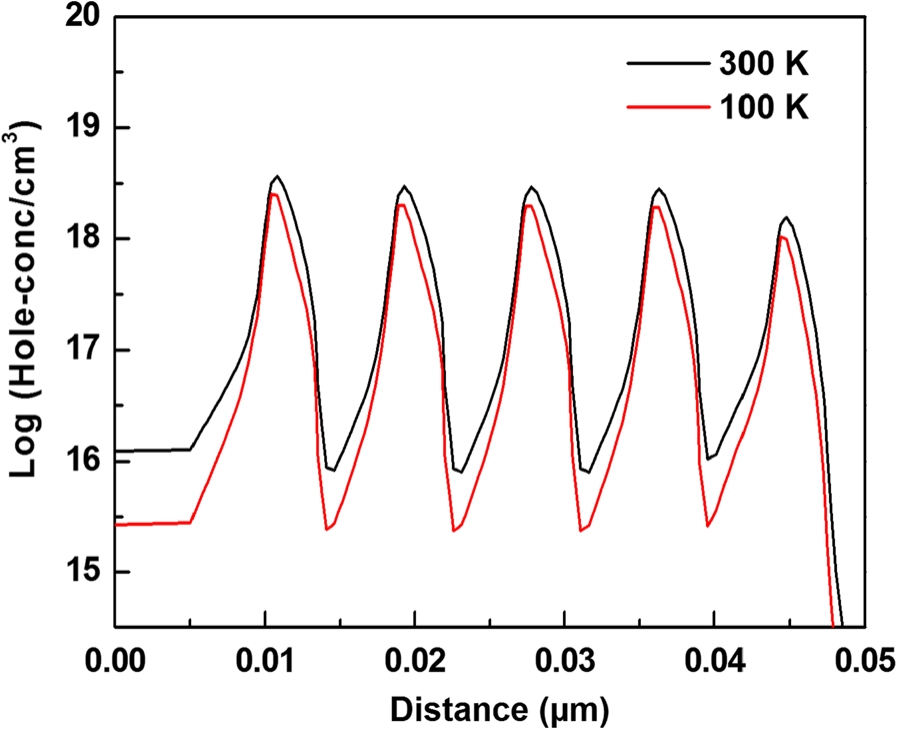
The hole concentrations in active region at 100 and 300 K for LED B under the injection of 100 mA

The EQE at low temperature was measured to evaluate the device performance. Figure 3a shows the relative EQE measured at 5 K. All the values were normalized to the maximum EQE of LED B. Obviously, the injection current for the maximum EQE decreased significantly compared to those at RT for all the devices. The maximum EQE of LED B was about 8.2 and 1.6 times than those of LEDs A and C, respectively. The current-dependent EQE were measured at different temperature. Figure 3b shows the current-dependent relative EQE at different temperature for LED B. All the values were normalized to the maximum EQE at 10 K. It can be seen that the current for the maximum EQE decreased as the temperature decreased. The same phenomenon was found for all three LEDs. It was well known that in bulk materials the hole concentration would decrease rapidly with decreasing temperature due to the high ionization energy of Mg in p-AlGaN. In our structure, it was demonstrated that the hole concentration also decreased as the temperature decreased [21]. We also simulated the hole distribution at different temperature. Figure 4 shows the hole concentrations in active region at 100 and 300 K for LED B under the injection of 100 mA. Obviously, the hole concentration decreased as the temperature decreased. In addition, the electron current overflowed out of QWs can be expressed as [22].

$$ {J}_{\mathrm{overflow}}=D{\left(\frac{\Delta E}{kT}\right)}^3 qBl $$

where *D* is a constant, Δ*E* is the difference of Fermi level and band edge of quantum wells, *K* is the Boltzmann constant, *T* is the temperature, *q* is the electron charge, *B* is the bimolecular radiative recombination coefficient, and *l* is the thickness of MQWs. For a certain LED, the contribution of variation of Δ*E* to *J*_overflow_ could be neglected compared to that of *T* as the temperature decreased. Therefore, the *J*_overflow_increased significantly at 5 K compared to that of RT, which was believed to be the main reason for the decrease of injection current at which the maximum EQE reached. The *J*_overflow_ decreased as the temperature increased, resulting in the increase of injection current for the maximum EQE, as shown in Fig. 3b. At low temperature, the internal efficiency would increase due to the freeze-out of nonradiative centers, such as the dislocations, which was beneficial to LED C with the largest volume of active region. This was the most possible reason why the EQE ratio of LED B to LED C decreased at 5 K compared to that at RT. Similarly, the EQE ratio of LED B to LED A increased at 5 K compared to that at RT.

## Conclusions

We studied the influence of QW width on EL properties of AlGaN DUV LEDs at different temperatures. The EL spectra showed a redshift as the QW width increased. The maximum EQE for LED with QW width of 3.5 nm was about 6.8 and 4.8 times than those of 2 and 5 nm at RT, respectively. However, these values changed to be 8.2 and 1.6 at 5 K, respectively. The different changes of maximum EQE ratios were attributed the decrease of non-radiative recombination and the increase of volume of the active region. From the theoretical analysis, the LED with 2-nm wells showed a shallowest barrier for electron overflow due to the quantum-confined effect, whereas the LED with 5-nm wells showed a least overlap of electron and hole due to the large internal field. Therefore, the LED with 3.5 nm QWs showed the highest maximum EQE. As the temperature decreased, the current for the maximum EQE decreased for all LEDs, which was believed to be due to the increase of electron overflowed out of QWs and the decrease of hole concentration. The maximum EQE for LED with QW width of 3.5 nm was about 8.2 and 1.6 times of those of 2 and 5 nm at 5 K, respectively, which was believed to be due to the decrease of non-radiative recombination centers and the increase of volume of active region.

## Abbreviations

DUV LEDs: Deep ultraviolet light-emitting diodes
EL: Electroluminescence
EQE: External quantum efficiency
MQW: Multiple quantum well
PL: Photoluminescence
QCSE: Quantum-confined Stark effect
QW: Quantum well
RT: Room temperature
